# Replication and Relevance of Multiple Susceptibility Loci Discovered from Genome Wide Association Studies for Type 2 Diabetes in an Indian Population

**DOI:** 10.1371/journal.pone.0157364

**Published:** 2016-06-16

**Authors:** Nagaraja M. Phani, Prabha Adhikari, Shivashankara K. Nagri, Sydney C. D’Souza, Kapaettu Satyamoorthy, Padmalatha S. Rai

**Affiliations:** 1 Department of Biotechnology, School of Life Sciences, Manipal University, Manipal-576104, Karnataka, India; 2 Department of Medicine, Kasturba Medical College, Manipal University, Mangalore-575001, Karnataka, India; 3 Department of Medicine, Kasturba Medical College, Manipal University, Manipal-576104, Karnataka, India; Children's National Medical Center, Washington, UNITED STATES

## Abstract

**Aim:**

Several genetic variants for type 2 diabetes (T2D) have been identified through genome wide association studies (GWAS) from Caucasian population; however replication studies were not consistent across various ethnicities. Objective of the current study is to examine the possible correlation of 9 most significant GWAS single nucleotide polymorphisms (SNPs) for T2D susceptibility as well as the interactive effect of these variants on the risk of T2D in an Indian population.

**Methods:**

Case-control cohorts of 1156 individuals were genotyped for 9 SNPs from an Indian population. Association analyses were performed using logistic regression after adjusting for covariates. Multifactor dimensionality reduction (MDR) analysis was adopted to determine gene–gene interactions and discriminatory power of combined SNP effect was assessed by grouping individuals based on the number of risk alleles and by calculating area under the receiver-operator characteristic curve (AUC).

**Results:**

We confirm the association of *TCF7L2* (rs7903146) and *SLC30A8* (rs13266634) with T2D. MDR analysis showed statistically significant interactions among four SNPs of *SLC30A8* (rs13266634), *IGF2BP2* (rs4402960), *HHEX* (rs1111875) and *CDKN2A* (rs10811661) genes. Cumulative analysis showed an increase in odds ratio against the baseline group of individuals carrying 5 to 6 risk alleles and discriminatory power of genetic test based on 9 variants showed higher AUC value when analyzed along with body mass index (BMI).

**Conclusion:**

These results provide a strong evidence for independent association between T2D and SNPs for in *TCF7L2* and *SLC30A8*. MDR analysis demonstrates that independently non-significant variants may interact with one another resulting in increased disease susceptibility in the population tested.

## Introduction

Type 2 Diabetes is phenotypically and genetically diverse and is a major global health concern affecting more than 76 million Indians and over 408 million individuals worldwide (http://www.idf.org/atlasmap/atlasmap). The risk of an individual towards T2D reflects the environmental influence in the background of genetic predisposition. Owing to the complex etiology of the disease progression, the identification of genetic markers has been slow until 2007. The advent of new technology in the form of microarray chips, has led to the development of high throughput genome wide association study (GWAS). Nevertheless, only a few variants in genes such as *KCNJ11*, *PPARG*, *SLC30A8*, and *TCF7L2* were reported to be linked with T2D [1–3]. However, GWAS in a French population by Sladek et al., 2007 reported variants in *TCF7L2*, *SLC30A8* and *HHEX* as new loci for T2D [4]. Subsequent GWAS in various populations have identified SNPs in several novel genes such as *IGF2BP2*, *PPARG*, *FTO*, *CDKN2A*, *CDKAL1*, *KCNQ1* and *JAZF1* to be associated with T2D [5–9]. Palmer et al., 2012 showed variants of *SLC44A3*, *RBM43*, *RND3*, *GALNTL4*, *TMEM45B* and *BARX2* as new susceptibility loci in African –American population [10]. Till date, GWAS have identified >70 susceptibility loci associated with T2D [10–19]. Besides, two well replicated genes, *PPARG* and *KCNJ11* which were initially shown to be associated with T2D through candidate gene studies were also confirmed through GWAS in European population [7, 9]. Though the association of many common variants established by GWAS has been replicated in several Caucasian populations, the results are conflicting in Asian population in general and Indian population in particular. Amongst all the association studies on T2D in Indian population, only the variants in *TCF7L2* (rs7903146, rs12243326 and rs4506565) has been consistently replicated and shown to be most promising [3, 20, 21].

Evidence from large population based and epidemiological studies showed that Indian population is genetically more prone to insulin resistance and diabetes [22]. Moreover, Indians develop T2D a decade earlier at a BMI which is much lower than Caucasians and this has been, attributed to their increased central obesity. This phenotype of increased tendency towards resistance of insulin effect is referred to as “Asian-Indian phenotype” [23]. Thus, understanding the contribution of the common genetic loci on T2D risk in Indian population comparatively to those originally identified by GWAS of European and American population is extremely important. However, there is a significant difference in the frequencies of risk alleles and linkage disequilibrium pattern in some genetic variants across different ethnicities [23] and hence there is a need to evaluate the same. Moreover, previous report of Indian Genome Variation (IGV) Consortium has showed how populations from Indian-sub-continent are discrete from HapMap populations [24]. Hence, in the present study we prioritized to evaluate previously identified common genetic variants in *KCNJ11* (rs5219), *TCF7L2* (rs7903146), *SLC30A8* (rs13266634), *IGF2BP2* (rs4402960), *HHEX* (rs1111875), *CDKN2A* (rs10811661), *KCNQ1* (rs2237892), *CDKAL1* (rs7754840) and *FTO* (rs8050136) for their association with T2D susceptibility. These SNPs have also been studied among Indian and non-Indian population with conflicting results. Our study population primarily comprises of a diverse mix of ethnic and linguistic groups of Karnataka state in southern part of India. Thus replication studies among the different ethnic and linguistic groups within the Indian subcontinent in general and within the south Indian population in particular may provide valuable information on the genetic risk factors for disease predisposition. Previous reports have shown that the cumulative effect of multiple common SNPs of small effect size are also likely to interact with each other in T2D [25]. Hence, the allele dosage and gene-gene interaction analysis of the variants was also performed to investigate their influence on T2D risk.

## Research Design and Methods

### Characteristics of study subjects

Study population comprised of 1156 unrelated individuals (578 cases and 578 controls) of southern part (Karnataka state) of India who were recruited from outpatient departments of Kasturba Hospital, Manipal and Mangalore, India. T2D subjects were recruited in accordance with following inclusion criteria: (1) T2D should be diagnosed according to world health organization (WHO) criteria, (2) onset of disease age should be >30 years and (3) should belong to south Indian origin. For control subjects, inclusion criteria’s were (1) levels of fasting glucose should be *<*126mg/dl and glycated haemoglobin (HbA1c) < 6.0%, (2) no history of diabetes in the first degree relatives, (3) age *>*40 years and (d) of south Indian origin. Written informed consent was obtained from all participating subjects and study was approved by the Institutional Ethics Committee, Kasturba Hospital, Manipal.

### Clinical and biochemical variables

General anthropometric measurements including height (m) and weight (kg) were obtained as per standardized protocols, and BMI was calculated using the formula weight (in kilograms)/height (in square meters). Fasting plasma glucose (FPG), total cholesterol (TC), high density lipoprotein (HDL) cholesterol, and triglycerides (TG) were estimated using Hitachi 912 auto-analyzer (Roche, Basel, Switzerland). Low density lipoprotein cholesterol (LDL) was calculated using the Friedewald formula (LDL = TC—HDL—TG/5.0 (mg/dl) and glycated haemoglobin (HbA1c) levels were estimated in whole blood using Cobas Integra 512 clinical chemistry auto-analyzer.

### SNP selection and Genotyping

Gene variants studied basically corresponds to those identified from GWAS of T2D till the year 2010 and also shown in replicative studies for its association with T2D susceptibility in other population. DNA was extracted from peripheral blood using standard phenol-chloroform procedure. A total of 9 SNPs [*KCNJ11* (rs5219), *TCF7L2* (rs7903146), *SLC30A8* (rs13266634), *IGF2BP2* (rs4402960), *HHEX* (rs1111875), *CDKN2A* (rs10811661), *KCNQ1* (rs2237892), *CDKAL1* (rs7754840) and *FTO* (rs8050136)] were genotyped in all the subjects. Genotyping of *KCNJ11* (rs5219), *FTO* (rs8050136) and*TCF7L2* (rs7903146) polymorphisms was carried out using Tetra primer amplification refractory mutation system (TETRA-ARMS) and genotyping of *SLC30A8* (rs13266634), *IGF2BP2* (rs4402960), *HHEX* (rs1111875), *CDKN2A* (rs10811661), *KCNQ1* (rs2237892) and*CDKAL1* (rs7754840) polymorphisms were carried out by polymerase chain reaction followed by restriction fragment length polymorphisms (PCR-RFLP). Random samples were picked and direct DNA sequencing was performed (Applied Biosystems USA 3130 genetic analyzer) to confirm the genotype.

### Statistical analysis

Quanto version 1.2.4 was employed for sample size calculation using minor allele frequency data from dbSNP database (http://www.ncbi.nlm.nih.gov/projects/SNP/). The categorical data of SNPs for association analysis with T2D was performed by using Pearson’s chi-squared test to detect differences in allele frequencies between cases and controls. Hardy–Weinberg equilibrium test was performed using a χ2 goodness-of-fit test to assess genotype frequencies. Association analysis was further confirmed by logistic regression after adjusting for age, sex and BMI as covariates and association results of SNPs with T2D was assessed by using odds ratio (OR) and corresponding 95% confidence interval (CI). In our analysis the most frequent homozygous genotype in the control population was considered as the reference category. Further we evaluated the differences in continuous variables (clinical variables between cases and controls) using Students t-test and data are represented as mean ± SD. Bonferroni correction has been used to reduce the chances of obtaining false-positive results (type I errors). A *P* value of less than 0.005 has been kept as a threshold of significance (after Bonferroni correction). All statistical analysis was performed using SPSS version 16 for windows (SPSS, Chicago, Illinois, USA) and SNPstat Software. Population attributable risk (PAR%) which identify what percentage of total risk for T2D is due to genetic effect of the variant was estimated for those SNPs which showed positive association with T2D susceptibility. For allele dosage analysis to acquire the combined information from multiple SNPs, we used allele count model where we summed the number of exact risk alleles carried by each individual in cases and control group.

### Multifactor dimensionality reduction (MDR) analysis

To investigate higher order gene-gene interaction among the tested SNPs, MDR (MDR, V2.0 Beta 2) method was employed. MDR is a nonparametric method and therefore no hypothesis concerning the value of any statistical parameter is made. It is also model free, thus no genetic inheritance model is assumed. All interactions and identification of best models were performed using 10 fold cross validation consistency (CVC) and permutation testing was done considering all possible SNP combination. Testing balance accuracy in the context of 10-fold cross-validation was used to assess model quality. An overall best model was selected that had the maximum accuracy in the testing data (i.e. testing accuracy or TA). We also recorded the cross-validation consistency or CVC. This provides a summary of the number of cross-validation intervals in which a particular model was found. Higher numbers indicate more robust results. Those models/SNP combinations with highest testing balanced accuracy (TBA) and CVC was selected as ‘‘best model’. This procedure generates an empirical estimate of the null distribution of testing accuracies and corrects for multiple testing because the same number of models are evaluated in all permutated and real data. All interactions were visualized by constructing an interaction dendrogram according to the method described by Moore et al., 2004 [26, 27, 28].

## Results

We genotyped 9 SNPs of GWAS in a case-control cohort of south Indian population consisting of 578 cases and 578 normal glucose tolerant (NGT) controls matched for age, sex and ethnicity. Table 1 shows the anthropometric and biochemical measurements among cases and NGT controls. In general, individuals with T2D had a higher BMI, HbA1c, FPG, TG, TC, LDL levels and lower HDL cholesterol levels. As shown in table 2, single marker association analysis for T2D revealed a significant association of *TCF7L2* (rs7903146) and *SLC30A8* (rs13266634) with an allelic OR = 1.46, 95% CI 1.15–1.85, *p* = 0.001 and OR = 1.33, 95% CI 1.10–1.60, *p* = 0.002. No significant association was observed between the remaining 7 loci and T2D in this population (*p*>0.05). Given that, variants in *FTO* and *HHEX* have shown to be associated with BMI, we further examined the association of these polymorphisms with BMI in our NGT control subjects and found that *FTO* (rs8050136) was significantly associated with BMI (Table 3).

**Table 1:** Clinical characteristics of study population.

Characteristics	T2D Subjects (n = 578)	Control Subjects (n = 578)	*P* value
**Age (Years)**	54.25 ±11.5	53.6 ±13.1	0.121
**Age at diagnosis (years)**	48.1 ± 10.6	-	-
**BMI (kg/m2)**	26.8 ± 3.4	22.8 ± 2.0	<0.05*
**HbA1c (%)**	8.55 ± 1.9	5.5 ± 1.6	<0.05*
**FPG (mg/dl)**	168.0 ± 58.9	88.7 ± 13.6	<0.05*
**TC (mg/dl)**	180.4 ± 37.3	169.2 ± 30.1	<0.05*
**TG(mg/dl)**	146.3 ± 49.3	102.7 ± 25.4	<0.05*
**HDL cholesterol (mg/dl)**	30.7 ± 11.1	46.6 ± 8.0	<0.05*
**LDL cholesterol (mg/dl)**	121.6 ± 36.5	108.0 ± 53.8	0.81

Data for quantitative variables are mean ± SD

*P value <0.05 is considered significant

Abbreviations: T2D-Type 2 diabetes, BMI-Body mass index, HbA1c-Glycated hemoglobin, FPG-Fasting plasma glucose, TC-Total cholesterol, TG-Triglycerides, HDL-High density lipoproteins, LDL-Low density lipoproteins.

**Table 2:** Genotype, allele distribution and association analysis of gene polymorphisms included in this study and risk of T2D under different genetic models.

Gene rsID	Allele and genotype	T2D (n = 578)	Control (n = 578)	OR (95% CI) *P-*Value			
				Allele	Codominant Model	Dominant Model	Recessive Model
***KCNJ11* rs5219**	C/T CC/CT/TT	746/410 254/238/86	745/413 255/235/89	0.99(0.83–1.17) *P* = 0.92	0.98(0.55–1.74) *P* = 0.84	0.94 (0.60–1.49) *P* = 0.8	1.13 (0.63–2.04)*P* = 0.69
***TCF7L2* rs7903146**	C/T CC/CT/TT	965/191 404/157/17	1020/138 450/120/9	1.46(1.15–1.85) *P = 0.001	0.39(0.18–1.06) *P* = 0.01	0.49(0.30–1.03) *P* = 0.005	0.32(0.07–1.48) *P* = 0.14
***SLC30A8* rs13266634**	C/T CC/CT/TT	822/334 293/236/49	887/271 339/209/31	1.33(1.10–1.60) *P = 0.002	0.68(0.36–1.32) *P* = 0.35	0.75(0.48–1.18) *P* = 0.21	0.63(0.27–1.48) *P* = 0.29
***IGF2BP2* rs4402960**	G/T GG/GT/TT	615/541 164/287/127	592/566 152/288/139	0.92(0.78–1.08) *P* = 0.31	1.39(0.76–2.54) *P* = 0.12	1.21(0.73–2.00) *P* = 0.46	1.78(1.03–3.07) *P* = 0.06
***HHEX* rs1111875**	C/T CC/CT/TT	897/259 340/217/21	879/279 333/213/33	0.91(0.75–1.10) *P* = 0.33	0.76(0.42–1.48) *P* = 0.13	1.02(0.64–1.61) *P* = 0.94	0.35(0.12–1.02)*P* = 0.05
***CDKN2A* rs10811661**	T/C CC/TC/TT	828/328 46/236/296	833/325 45/235/299	0.98(0.82–1.18) *P* = 0.86	1.05(0.56–2.01) *P* = 0.74	1.13(0.71–1.79) *P* = 0.61	0.86(0.39–1.89) *P* = 0.7
***KCNQ1* rs2237892**	C/T CC/CT	1088/68 510/68	1093/65 514/65	1.05(0.74–1.49) *P* = 0.78	NA	NA	NA
***CDKAL1* rs7754840**	G/C CC/GC/GG	1000/156 15/126/437	984/174 12/150/417	1.13(0.89–1.43) *P* = 0.29	0.47(0.24–01.20) *P =* 0.12	0.68(0.41–1.14) *P* = 0.14	0.22(0.04–1.25) *P* = 0.08
***FTO* rs8050136**	A/T AA/AT/TT	655/501 189/277/112	629/529 176/277/126	0.90(0.77–1.07) *P* = 0.25	1.61(0.91–2.86) *P* = 0.14	1.61(1.0–2.58) *P* = 0.069	1.21(0.69–2.14) *P* = 0.5

*P value <0.05 is considered significant, 95% CI that did not include unity is statistically significant

*P* values were adjusted for age, gender and BMI.

**Table 3:** Association of the SNP loci with BMI in control subjects.

SNP/Gene	Genotype(n)/BMI			*P* value
***KCNJ11* rs5219**	CC (255) 23.01±1.89	CT (235) 22.71±2.08	TT(89) 23.08 ±2.07	0.24
***TCF7L2* rs7903146**	CC (450) 22.87±1.97	CT (120) 22.9±2.16	TT (9) 23.8±0.84	0.75
***SLC30A8* rs13266634**	CC (339) 22.91±2.0	CT (209) 22.89±2.0	TT (31) 22.64±1.85	0.79
***IGF2BP2* rs4402960**	GG (152) 22.92±1.87	GT (288) 23.08±2.06	TT (139) 22.71±1.92	0.27
***HHEX* rs1111875**	CC (333) 22.87±1.98	CT (213) 22.84±2.05	TT (33) 23.16±2.03	0.70
***CDKN2A* rs10811661**	CC (45) 23.10±1.99	TC (235) 22.71±2.12	TT (299) 22.96±1.92	0.34
***CDKAL1* rs7754840**	CC (12) 22.84±1.97	GC (150) 23.02±2.03	GG (417) 22.72±2.38	0.64
***FTO* rs8050136**	AA (176) 21.99±2.15	AT (277) 22.65±1.92	TT (126) 23.32±1.95	*0.01

Values are mean ± SD and were compared using ANOVA

* *P<*0.05 is considered statistically significant

Abbreviations: BMI- Body mass index

**Fig 1:**
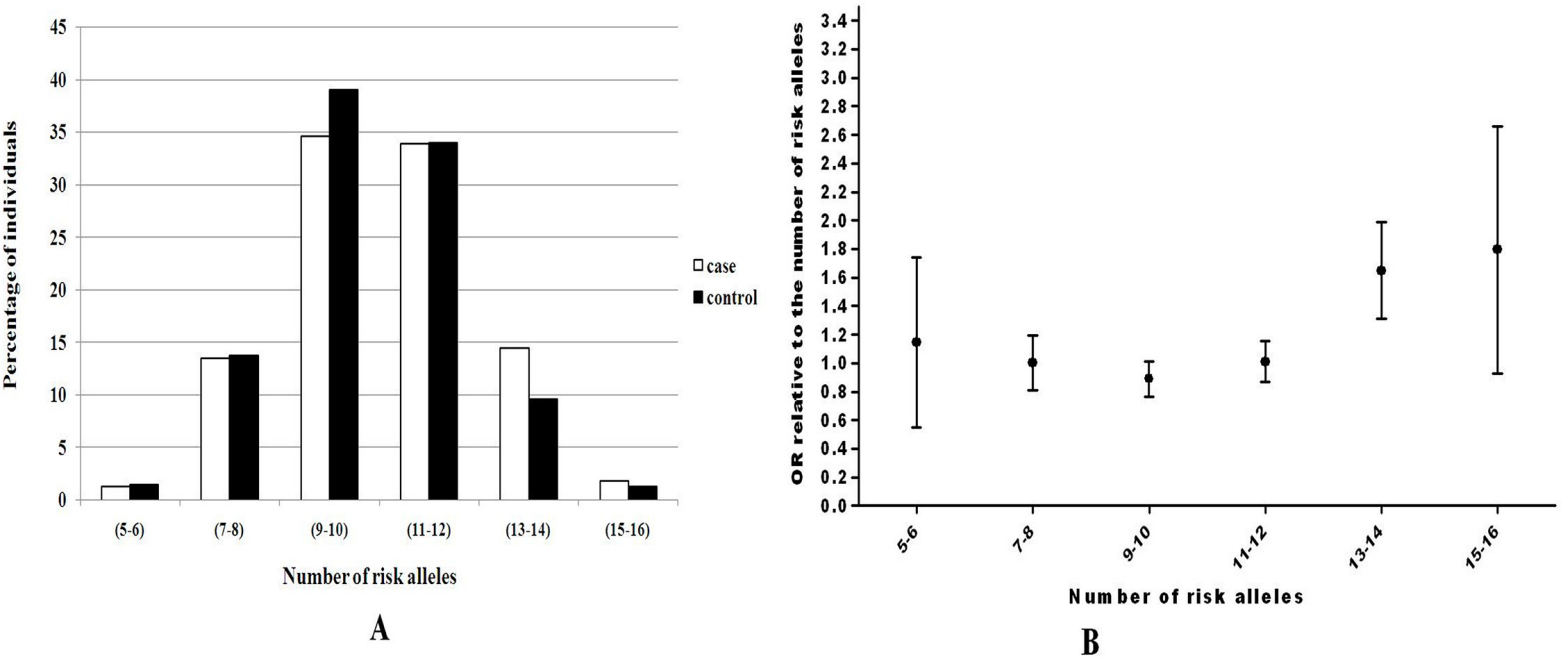
(a) Distribution of risk alleles in type 2 diabetic (white bars) and control subjects (black bars) (b) Depiction showing the trend in ORs with the increasing number of type 2 diabetes risk alleles versus the baseline of 5–6 risk alleles. The vertical bars represent 95% CIs.

**Fig 2:**
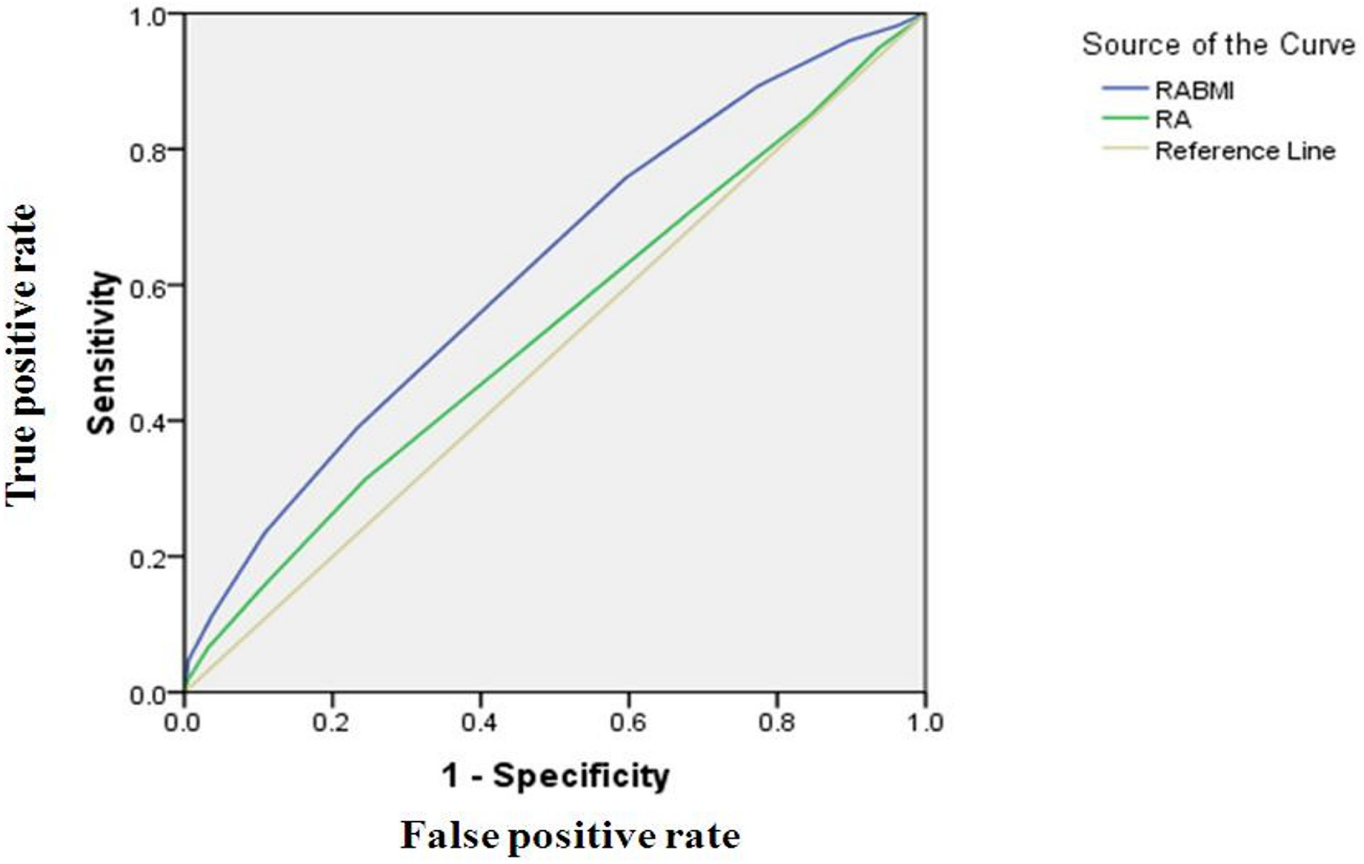
ROC plot for 9 variants alone (AUC -0.536) and 9 variants plus BMI (AUC- 0.624).

The individual risk variants in our study showed similar effect sizes as compared to other large studies from Caucasian population (2, 4, 5, 7, 9). Several variants in our study though are not associated with T2D at *p*<0.05 were still included in our allele dosage analysis because these were confirmed T2D risk variants and lack of significance may probably due to lower risk allele frequencies. Fig 1 represents the percentage of cases and NGT control subjects who were grouped according to the number of exact risk alleles they carry. Risk allele distribution followed a normal distribution pattern in cases and NGT controls with a shift towards higher risk allele number in T2D subjects. There was an increase in OR for T2D with an increase in the number of risk alleles against the baseline group of 1.3% of individuals carrying exactly 5–6 risk alleles. Of individuals with exactly 13–14 risk alleles 14.5% had an OR of 1.58 (95% CI 1.1–2.27) against the baseline reference group. We further evaluated the percentage of total risk for T2D and genetic effect of *SLC30A8* (rs13266634) and *TCF7L2* (rs7903146) which were found to be associated with T2D in our population. We observed that *TCF7L2* (rs7903146) accounts for 18.4% and *SLC30A8* (rs13266634) accounts for 30.4% PAR. These two genetic regions contribute to a total of 48.8% population attributable risk (PAR) for T2D (Table 4). We assessed the discriminatory power of genetic test based on nine T2D variants by calculating the area under ROC (Receiver operating characteristic) curve (Fig 2). The area under ROC curve for nine T2D variants studied here was 0.536. Similar analysis was performed for obese and non-obese subgroups in the cohort which showed an area under the curve value of 0.514 and 0.529 respectively. Further, we tested if BMI would add to discriminatory power of these 9 risk variants and the area under the curve showed marginal increase (0.624) when BMI and risk variants were assessed cumulatively. No significant difference in area under the curve was observed when *FTO* (rs8050136) was removed from analysis.

**Table 4:** Comparison of risk allele frequency and Population-Attributable Risk (PAR) between South Indian and Caucasian populations.

Gene rsID	T2D Risk allele in South Indian population	T2D Risk allele in Caucasian population	Risk allele frequency		Population Attributable Risk (%)	
			Current study	Caucasian population	Caucasian population	Current Study
***TCF7L2* rs7903146**	T	T	0.14	0.29	18.4	15.0
***SLC30A8* rs13266634**	T	C	0.26	0.69	30.4	14.6

Caucasian allele frequency obtained from Alfred data base (http://alfred.med.yale.edu/) accessed on: 4^th^ April 2015

**Fig 3:**
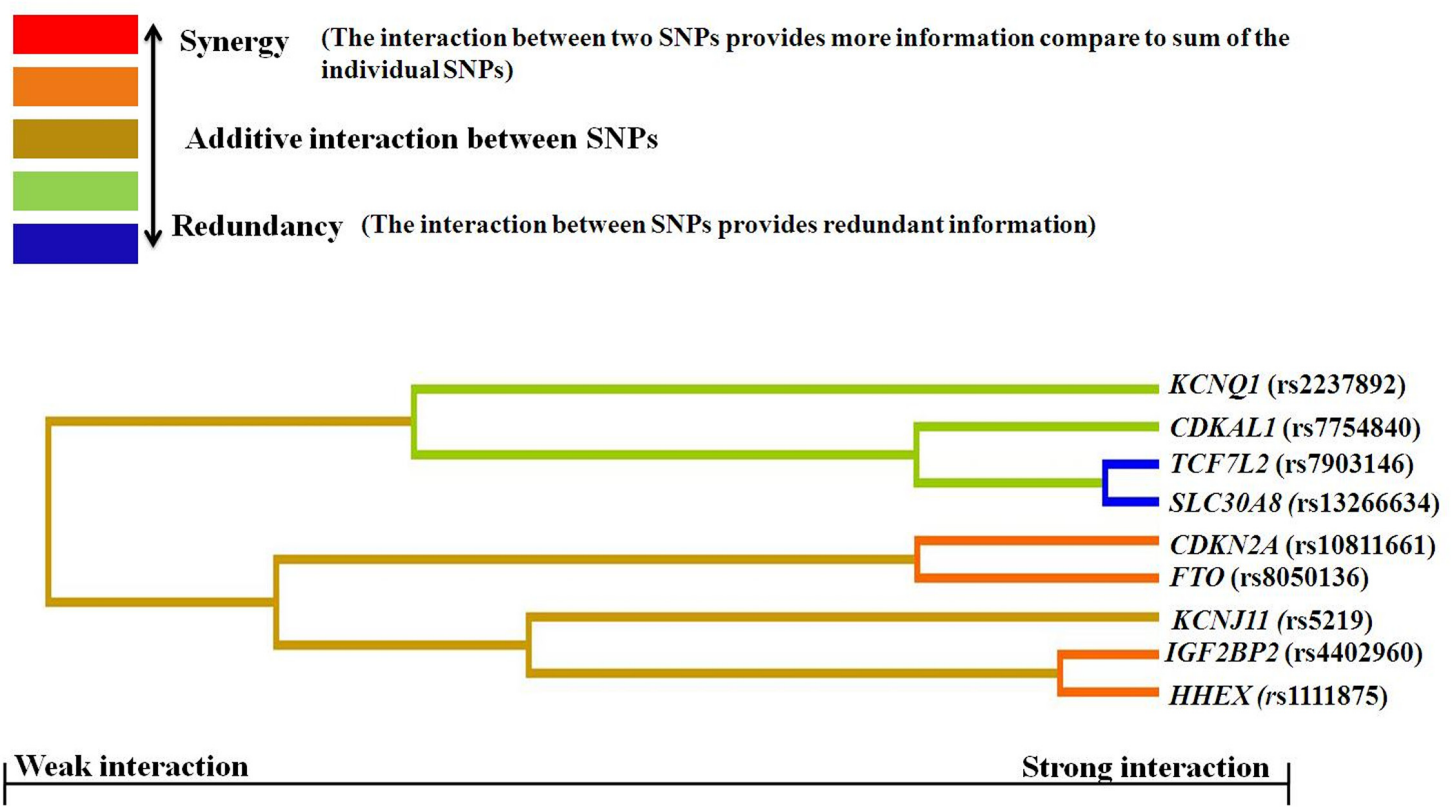
The dendrogram representing the nature of the interactions between SNPs.

**Fig 4:**
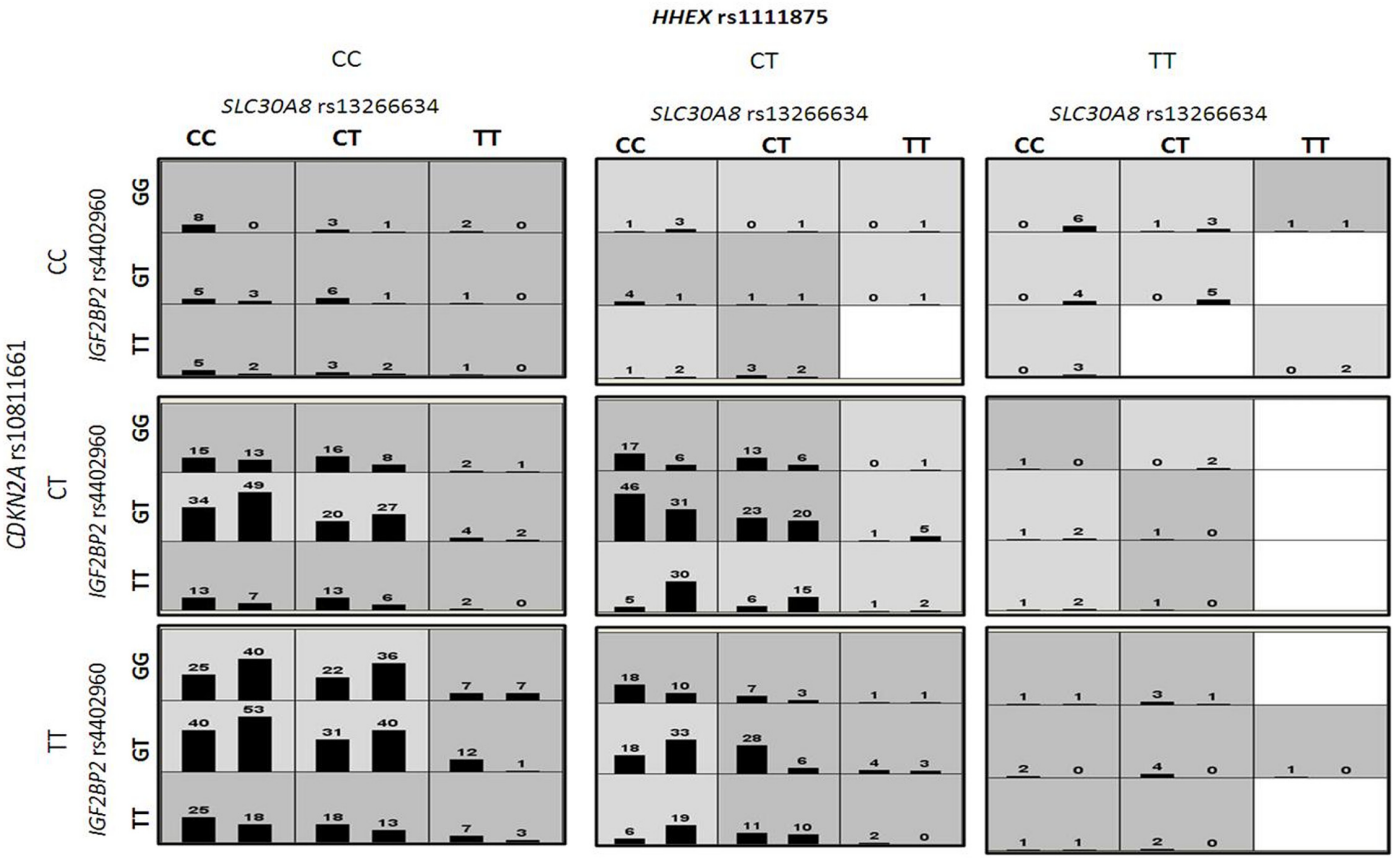
The best four-locus SNP model selected by MDR. Interaction of *SLC30A8* (rs13266634), *IGF2BP2* (rs4402960), *HHEX* (rs1111875) and *CDKN2A* (rs10811661) polymorphisms is shown. Cells shaded in dark represents higher risk groups and lighter shaded cells represents lower risk groups. White cells or no shaded cells represent genotype combination where no data are available. Bars represent hypothetical distributions of cases (left) and controls (right) with each multifactor combination.

Consecutively to expand the findings in our data, MDR analysis was applied to detect higher order gene-gene interaction in cases and controls. MDR analysis of the above mentioned variants revealed statistically significant interactions. Best interaction models along with testing accuracy and cross validation consistency are represented in table 5. The overall best model with highest CVC consisted of a model that included *SLC30A8* (rs13266634), *IGF2BP2* (rs4402960), *HHEX* (rs1111875) and *CDKN2A* (rs10811661) SNPs. This model has a significant TBA of 0.618 and a CVC of 10/10. However it’s worth mentioning that 3 SNPs showing genetic interaction in this model were not associated with increased risk of T2D in univariate analysis (*IGF2BP2* (rs4402960), *HHEX* (rs1111875) and *CDKN2A* (rs10811661)). The entropy based dendrogram obtained by MDR evidently showed an intricate and a hierarchical pattern of interaction among the gene variants constituting the polygenic basis of the disease (Fig 3). In particular *CDKN2A* (rs10811661) and *FTO* (rs8050136), *IGF2BP2*(rs4402960) and *HHEX* (rs1111875) loci showed maximum degree of synergy in their interactions and on the contrary, different degree of redundancy (antagonism) was observed between *TCF7L2* (rs7903146) and *SLC30A8* (rs13266634). Fig 4 depicts a graphical representation of the combined effect of four locus model of *SLC30A8* (rs13266634), *IGF2BP2* (rs4402960) and *HHEX* (rs1111875), and *CDKN2A* (rs10811661) as high and low risk groups along with its statistical interactions. As per the four locus model, individuals homozygous for the wild type allele in *SLC30A8* (rs13266634), *IGF2BP2* (rs4402960), *HHEX* (rs1111875) and individuals homozygous for the mutant allele in *CDKN2A* (rs10811661) were placed in low risk group while, individuals heterozygous for all four SNPs were placed in high risk group. However, we also observed that individuals heterozygous for at least three of the four alleles were also in high risk group.

**Table 5:** Multifactor dimensionality reduction analysis summary.

Interacting SNPs	CVC	TBA	*P* value
***IGF2BP2* rs4402960,*HHEX* rs1111875**	10/10	0.564	0.160
***IGF2BP2* rs4402960, *HHEX* rs1111875, CDKN2A rs10811661**	10/10	0.606	0.021
**SLC30A8 rs13266634, *IGF2BP2* rs4402960, *HHEX* rs1111875, *CDKN2A* rs10811661**	10/10	0.618	0.011*
***KCNJ11* rs5219, *SLC30A8* rs13266634, *IGF2BP2* rs4402960, *HHEX* rs1111875, *CDKN2A* rs10811661, *KCNQ1* rS2237892, *FTO* rs8050136**	10/10	0.542	0.338
***KCNJ11* rs5219, *SLC30A8* rs13266634, *IGF2BP2* rs4402960, *HHEX* rs1111875, *CDKN2A* rs10811661, *CDKAL1* rs7754840, *KCNQ1* rS2237892, *FTO* rs8050136**	10/10	0.542	0.319

**CVC-**Cross Validation Consistency, **TBA-**Testing Balanced Accuracy

*P value <0.05 is considered significant

## Discussion

The outcome of GWAS has significantly contributed to the discovery of number of genetic loci linked to T2D [10–19]. In the present investigation, we have performed an unbiased replication study of SNPs associated with T2D derived from previously published GWAS in our independent dataset from south Indian origin. One of the selected SNP (*KCNJ11* rs5219) belongs to well replicated biological candidate gene which has shown a clear evidence of its relationship to T2D susceptibility [2, 3]. Until now, only *TCF7L2* (rs7903146) has been shown to have a strongest association with the highest effect size for T2D association across various ethnicities [3, 4, 5, 15, 29, 30]. The current study confirms the association of *TCF7L2* (rs7903146) with T2D susceptibility with an effect size of 1.46, 95% CI 1.15–1.85, *p* = 0.001. Sladek et al., 2007 was the first to identify *SLC30A8* (rs13266634) as a novel gene variant associated with T2D and since then, many studies have been performed across various ethnic population but the results have been contradictory [4,7,29,31]. Though previous reports from Omori et al., 2008, Wu et al., 2008 and Ng et al., 2008 from Asian populations showed a positive association, these results were not replicated by Sanghera et al., 2008 in an Indian Sikh population which may be attributed to lower sample number and low power of the study [32–34,29]. Subsequently, a large scale study by Chauhan et al., 2010 in northern and western Indian population showed a strong association of this polymorphism with T2D [21]. In the present study, we found a positive association of this polymorphism with T2D with an effect size of 1.33, 95% CI 1.10–1.60, *p* = 0.002.

While the significance of *SLC30A8* and *TCF7L2* as a key gene for T2D susceptibility has been well known, the genetic variants in these genes could account for about ~20% of all T2D cases in the Caucasian population [35, 36]. The PAR of *SLC30A8* and *TCF7L2* polymorphisms in Caucasian population was 30.4% and 18.4% respectively which is much higher when compared to our population, and this could be attributed to the higher risk allele frequency in Caucasian population. Further in our allele dosage analysis, we have identified a particular subset of individuals at different risk of disease by weighing against the individuals with smallest number of risk alleles in comparison to those with the majority of risk alleles. This in turn allowed us to identify subgroups of the population with distinctly differing risk for disease. In our population, we were able to distinguish 14.5% of individuals carrying >13–14 risk alleles that had four times increased risk of T2D when compared to 1.3% of individuals with >5–6 risk alleles. The high risk group also had over thrice the OR for T2D than those with the lower number of risk alleles. Thus, variants are not predominantly discriminative but only explain a small percentage of heritability of T2D. However, instead of focusing on individuals with elevated risk alleles at the population level, the efficacy of genetic test perhaps is better utilized by means of ROC curves. The nine T2D variants though had an insufficient discriminatory ability with an AUC of 0.536; a marginal increase was obtained when it was analyzed with BMI with an AUC value of 0.624.

Another major finding of our study was the establishment of gene interaction model between *SLC30A8*, *IGF2BP2*, *HHEX*, and *CDKN2A* genes towards T2D susceptibility by MDR analysis. Among the five models for all the loci tested (nine SNPs of nine candidate genes), the best gene-gene interaction model identified was a four locus model which includes *SLC30A8* (rs13266634), *IGF2BP2* (rs4402960), *HHEX* (rs1111875) and *CDKN2A* (rs10811661). Individuals heterozygous for at least three out of four loci were shown to cluster in high risk groups. In this model the combination of *HHEX* (rs1111875) CC homozygotes, *SLC30A8* (rs13266634) CC homozygotes, *IGF2BP2* (rs4402960) GG homozygotes and *CDKN2A* (rs10811661) TT homozygotes were associated with reduced risk to T2D. Our study did not show any independent association of *IGF2BP2* (rs4402960), *HHEX* (rs1111875) and *CDKN2A* (rs10811661) with T2D. However in MDR analysis, combined effect of these polymorphisms with *SLC30A8* (rs13266634) was associated with increased risk of T2D which suggest a cross talk between these genes in the pathogenesis of T2D. Further, a synergistic interaction among these SNPs suggests a significant role of epistasis in the susceptibility of T2D. However, it is also observed that all the SNPs which were included in this study showed a weak synergistic effect with each other and this suggests the interaction of susceptibility alleles with environmental and life style factors. Nevertheless, the risk allele frequencies of these variants in the tested population were entirely different with those from Caucasian population and thus, the absence of positive association towards T2D in remaining loci points towards the contribution of these polymorphisms to T2D susceptibility is not convincing across different ethnicities or to a particular genetic background. To the best of our knowledge, this is the first report from south Indian population trying to investigate the role of these polymorphisms individually and cumulatively towards T2D susceptibility. Nonetheless, further replication studies are warranted to validate these and newer susceptibility loci which are identified from large scale GWAS in independent samples collected from other ethnic groups.

## Supporting Information

S1 Table List of genotype and clinical characteristics of study subjects. (XLSX)
